# Effect of pre-analytic variables on the reproducibility of qPCR relative telomere length measurement

**DOI:** 10.1371/journal.pone.0184098

**Published:** 2017-09-08

**Authors:** Casey L. Dagnall, Belynda Hicks, Kedest Teshome, Amy A. Hutchinson, Shahinaz M. Gadalla, Payal P. Khincha, Meredith Yeager, Sharon A. Savage

**Affiliations:** 1 Division of Cancer Epidemiology and Genetics, National Cancer Institute (NCI), Bethesda, Maryland, United States of America; 2 Cancer Genomics Research Laboratory, Leidos Biomedical Research, Inc., Frederick National Laboratory for Cancer Research, Frederick, Maryland, United States of America; Centre National de la Recherche Scientifique, FRANCE

## Abstract

Telomeres, long nucleotide repeats and a protein complex at chromosome ends, shorten with each cell division and are susceptible to oxidative damage. Quantitative PCR (qPCR) is a widely-used technique to measure relative telomere length (RTL) in DNA samples but is challenging to optimize and significant lab-to-lab variability has been reported. In this study, we evaluated factors that may contribute to qPCR RTL measurement variability including DNA extraction methods, methods used for removing potential residual PCR inhibitors, sample storage conditions, and sample location in the PCR plate. Our results show that the DNA extraction and purification techniques, as well as sample storage conditions introduce significant variability in qPCR RTL results. We did not find significant differences in results based on sample location in the PCR plate or qPCR instrument used. These data suggest that lack of reproducibility in published association studies of RTL could be, in part, due to methodological inconsistencies. This study illustrates the importance of uniform sample handling, from DNA extraction through data generation and analysis, in using qPCR to determine RTL.

## Introduction

Telomeres are comprised of (TTAGGG)_n_ nucleotide repeats and a protein complex that protect chromosome ends [1]. They shorten with each cell division due to the inability of DNA polymerase to replicate the 3’ end of DNA. Telomere length (TL) in blood or buccal cell DNA has been associated with cancer, heart disease, and several other illnesses [2–10].

Numerous TL measurement methods exist, each with advantages and limitations [11, 12]. PCR-based methods to measure TL are widely used because they require small amounts of DNA and are often less labor intensive than other methods. There are currently three reported methods to perform PCR analysis of TL: quantitative PCR (qPCR), monochrome multiplex qPCR (MMqPCR), and absolute telomere length qPCR (aTLqPCR). The first, qPCR, utilizes primers targeting the telomeric hexanucleotide repeats [13]. In the qPCR method, two separate qPCR reactions are performed and the resulting amount of telomere amplicons (T) are compared to the amount of a single-copy gene amplicons (S) to generate the T/S ratio, resulting in a relative TL (RTL) rather than an absolute measure. The MMqPCR method performs both the T and the S reactions in the same well, thus reducing pipetting precision as a variable [14]. The aTLqPCR method adapted the original qPCR method to determine a base pair estimate of average TL by using a standard curve of synthesized telomeric repeat sequence oligonucleotide diluted to a known TL [15].

The original qPCR method, published in 2002, is the most widely used in large-scale studies, but has known variability within and between batches and lacks reference standards that are necessary to ensure consistency of results [11]. The correlation between qPCR RTL with other TL measurement methods, including terminal restriction fragment analysis (TRF) and flow cytometry with fluorescent *in situ* hybridization (flow FISH), varies with correlation coefficients (R^2^) ranging from 0.1 to 0.99 [13, 16–20]. Many association studies using qPCR RTL measurement have not reported important details, such as DNA extraction methods, specific reagents and single copy loci used, as well as method of RTL value generation [12]. Others have shown that the DNA extraction method [21–26], tissue fixation method [27], and well position [28] are possible sources of variability in qPCR RTL measurement.

To address the factors contributing to qPCR RTL variability, we comprehensively evaluated the effects of DNA extraction method, PCR inhibitor removal methods, sample storage conditions, and sample location in the PCR plate.

## Materials and methods

### DNA extraction methods

Buffy coat specimens from 48 subjects, in the Research Donor Program at the Frederick National Laboratory for Cancer Research, were mixed thoroughly and split into three equal volume aliquots. Each homogenous aliquot was then extracted via QIAamp DNA Blood Midi Kit (Qiagen, Germantown, MD), QIAsymphony DNA Midi Kit (Qiagen), and ReliaPrep Large Volume HT gDNA Isolation System (Promega, Madison, WI). The QIAsymphony and ReliaPrep kits utilize magnetic bead/particle-based methods, while the QIAamp kit uses silica-membrane-based nucleic acid purification method. The DNA was quantified with Quant-iT PicoGreen dsDNA quantitation (Life Technologies, Grand Island, NY).

### qPCR relative telomere length assay

DNA samples were transferred into 96-well plates and the concentration normalized to 1 ng/uL. We also randomly placed no template control (NTC) and internal quality control (QC) sample replicates, NA07057 (Coriell Cell Repositories, Camden, NJ), as calibrator samples. Four uL of DNA (4 ng) was then transferred, in triplicate, into quadrants 1, 2, and 3 of LightCycler-compatible 384-well plates (Roche, Indianapolis, IN) and a standard curve [6 concentrations of pooled reference DNA samples prepared by serial dilution (4 to .04096 ng/uL)] was added to quadrant 4 of each 384-well plate, all samples were dried down. This resulted in all experimental and control samples being assayed in triplicate on each 384-well plate for both T and S assays. All pipetting steps were performed using a Biomek FX (Beckman Coulter, Indianapolis, IN) liquid handler calibrated to perform transfers from 2–50 uL with a coefficient of variation (CV) of <5%.

Primers for the telomeric assay were *Telo_FP* [5’-CGGTTT (GTTTGG)_5_GTT-3’] and *Telo_RP* [5’-GGCTTG (CCTTAC)_5_CCT-3’] [29] and for the single-copy gene (36B4) assay were *36B4_FP* [5’-CAGCAAGTGGGAAGGTGTAATCC-3’] and *36B4_RP* [5’-CCCATTCTATCATCAACGGGTACAA-3’] [13]. Primers (Integrated DNA Technologies, Coralville, IA) were manufactured LabReady (normalized to 100 uM in IDTE, pH 8.0 and HPLC Purified). One μM assay mixes for each target were generated by combining 990 uL of 1X Tris-EDTA Buffer with 5 uL of forward oligo and 5 uL of reverse oligo.

PCR was performed using 5 uL reaction volumes consisting of: 2.5 uL of 2X Rotor-Gene SYBR Green PCR Master Mix (Qiagen), 2.0 uL of MBG Water, and 0.5 uL of 1 μM assay-specific mix. Thermal cycling was performed on a LightCycler 480 (Roche) where PCR conditions were (*i)* T (telomeric) PCR: 95°C hold for 5 minutes (min), denature at 98°C for 15 seconds, anneal at 54°C for 2 min, with fluorescence data collection, 35 cycles and (*ii)* S (single-copy gene, 36B4) PCR: 98°C hold for 5 min, denature at 98°C for 15 seconds, anneal at 58°C for 1 min, with fluorescence data collection, 43 cycles.

LightCycler software (Release 1.5.0) was used to generate Ct values, utilizing absolute quantification analysis with the second derivative maximum method and high sensitivity detection algorithm. Ct values or replicates were averaged, if they met a coefficient of variation (CV) threshold of less than 2%. The concentration (ng/uL) was interpolated from the plate-specific standard curve’s exponential regression [Average Ct and log2 (Concentration)]. Any samples with 36B4 concentrations falling outside the range of the standard curve are dropped from further analysis as a T/S ratio cannot be accurately calculated. The telomere (T) concentration was divided by the 36B4 concentration (S) to yield a raw T/S ratio. The raw T/S ratio is divided by the average raw T/S ratio of the internal QC calibrator samples, within the same plate set, to yield a standardized T/S ratio that normalized results in reference to the same individual.

### Evaluation of assay reproducibility

A single sample, the internal QC calibrator sample, was diluted to 1 ng/uL and then aliquoted into every well of a 96-well intermediate plate. This intermediate plate was used to aliquot this single sample, in triplicate, to twelve 384-well assay plates. Six assay plates were prepared with the Telomere assay and six with the 36B4 assay. Two plates for each assay were thermal cycled on three different LightCyclers.

### DNA purification

After determining the baseline RTL, we applied three different DNA purification methods on 30 DNA samples from 10 subjects, extracted as described above (3 DNA samples/subject using different extraction techniques). The 30 DNA samples were mixed thoroughly and three 500 ng aliquots were created, which were purified using ethanol (EtOH) precipitation, MinElute (Qiagen), a silica-membrane-based purification, and AMPureXP (Beckman Coulter), a magnetic bead-based DNA capture method, creating 90 samples for qPCR analysis.

### DNA storage temperature and concentration

We determined the baseline RTL of 50 different DNA samples and then subjected aliquots of these samples to different storage conditions for 6 months: 4°C at 25 ng/uL, 4°C at 1 ng/uL, -30°C at 25 ng/uL, and -30°C at 1 ng/uL.

## Results

### Well position and assay reproducibility

We first evaluated the reproducibility of the qPCR assay by measuring RTL on a single DNA sample aliquoted into a 96-well intermediate plate, then into 384-well plates for qPCR (S1 Dataset). The average amplification efficiency for the Telo assay was 96.66%, with a CV of 0.14% and for the 36B4 assay was 97.44%, with a CV of 0.25%. The average standardized T/S ratio for all RTL results (n = 576) was 1.00 and the CV was 2.20%. Three different Light Cyclers were used to determine whether there was machine-to-machine variability. We ran 192 replicates on each LightCycler and found average standardized T/S ratios of 0.99, 1.00, and 1.00 with respective CVs of 2.58%, 1.79%, and 2.17%. Overall, the RTL results were reproducible and had little variability both within and across plates, run on various instruments.

### Evaluation of DNA extraction method

**Fig 1:**
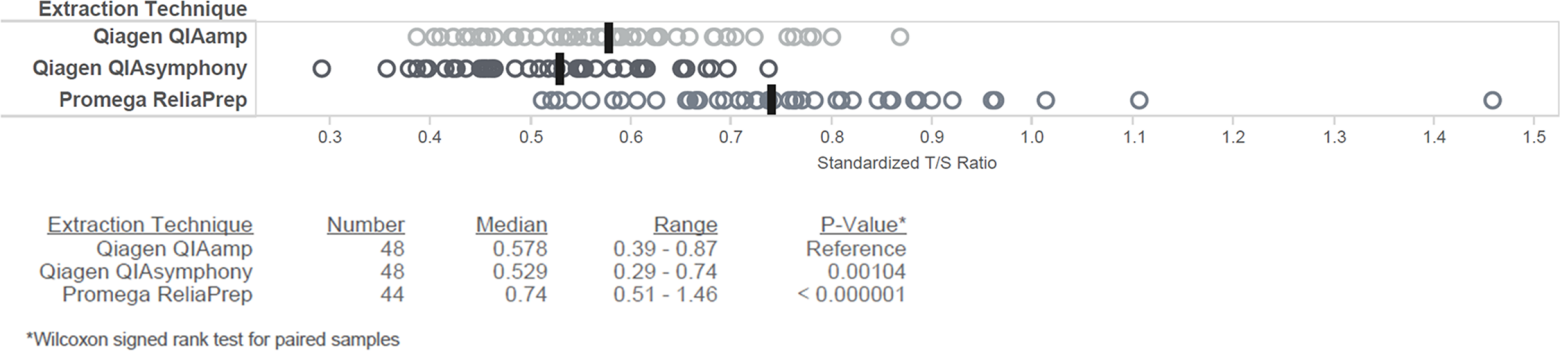
Extraction techniques contribute to differences in dynamic range of relative telomere length. (Top) Dynamic range of RTL (standardized T/S ratio) by extraction technique in matched samples from the same subjects, median marked by black bar and (Bottom) count of samples, median standardized T/S ratio, range of standardized T/S ratio, and p-value for Wilcoxon signed rank test for paired samples by each extraction technique.

**Fig 2:**
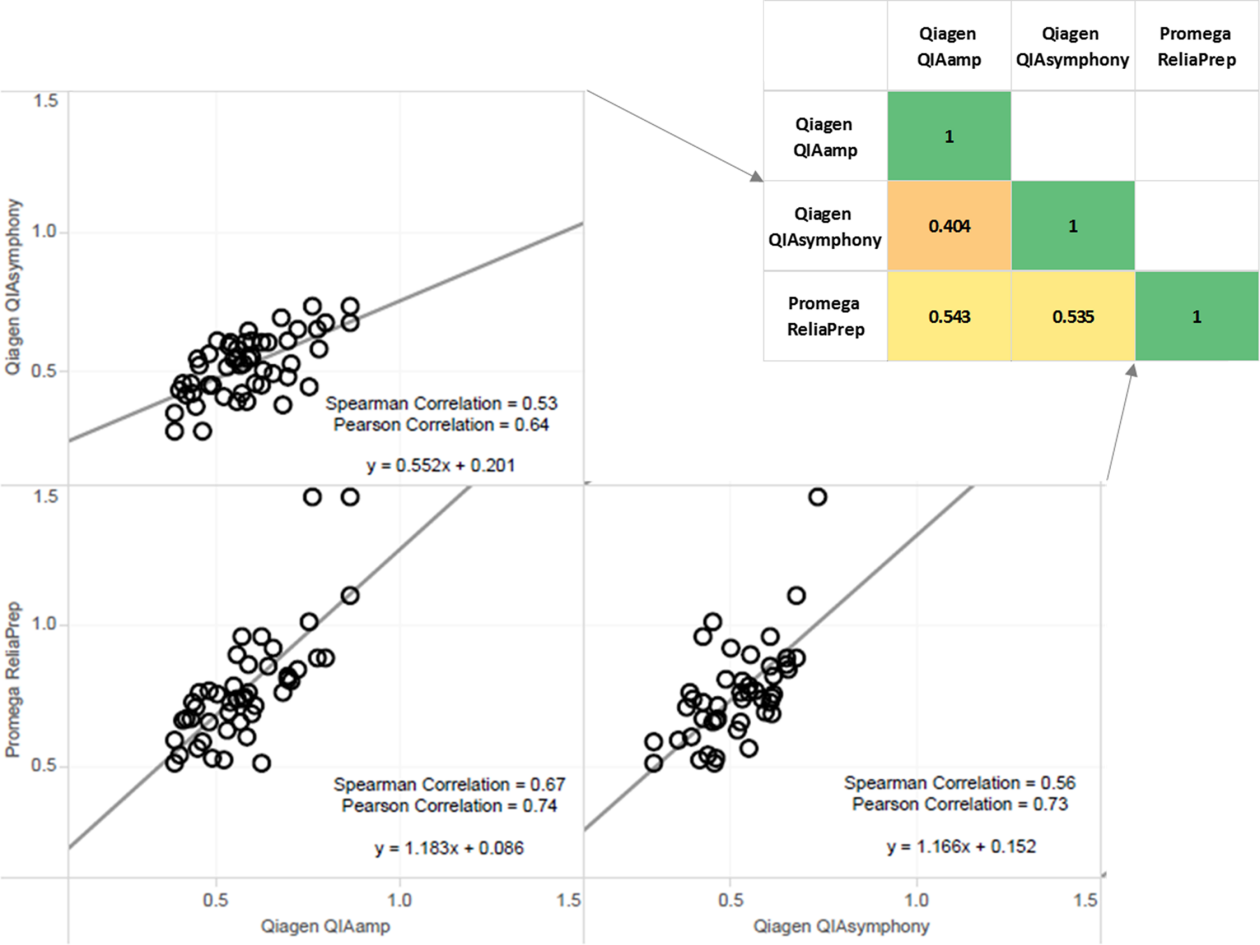
Correlation of relative telomere length (standardized T/S ratio) of matched subjects across extraction techniques and assay techniques. Inset heat map displays coefficient of determination (R^2^) for each correlation.

The median and range of RTL of buffy coat DNA varied by extraction method (Fig 1, S2 Dataset). The QIAamp RTL median T/S ratio was 0.58 (range 0.39–0.87). QIAsymphony median T/Swas 0.53 (range 0.29–0.74) and the ReliaPrep median was 0.74 (range 0.51–1.46). The median RTL differences between QIAamp and QIAsymphony or ReliaPrep, as determined by Wilcoxon signed rank test for paired samples, were statistically significant (p = 0.001, <0.001, respectively). For these samples, the CV for internal control replicates (n = 41) standardized T/S ratio was 5.13%. Correlation of RTL for the 48 matched subjects between extraction methods was modest (R^2^ = 0.40, 0.54, and 0.54) as was Spearman’s rank-order correlation (ρ = 0.53, 0.67, and 0.56) (Fig 2).

### Evaluation of DNA purification techniques

**Fig 3:**
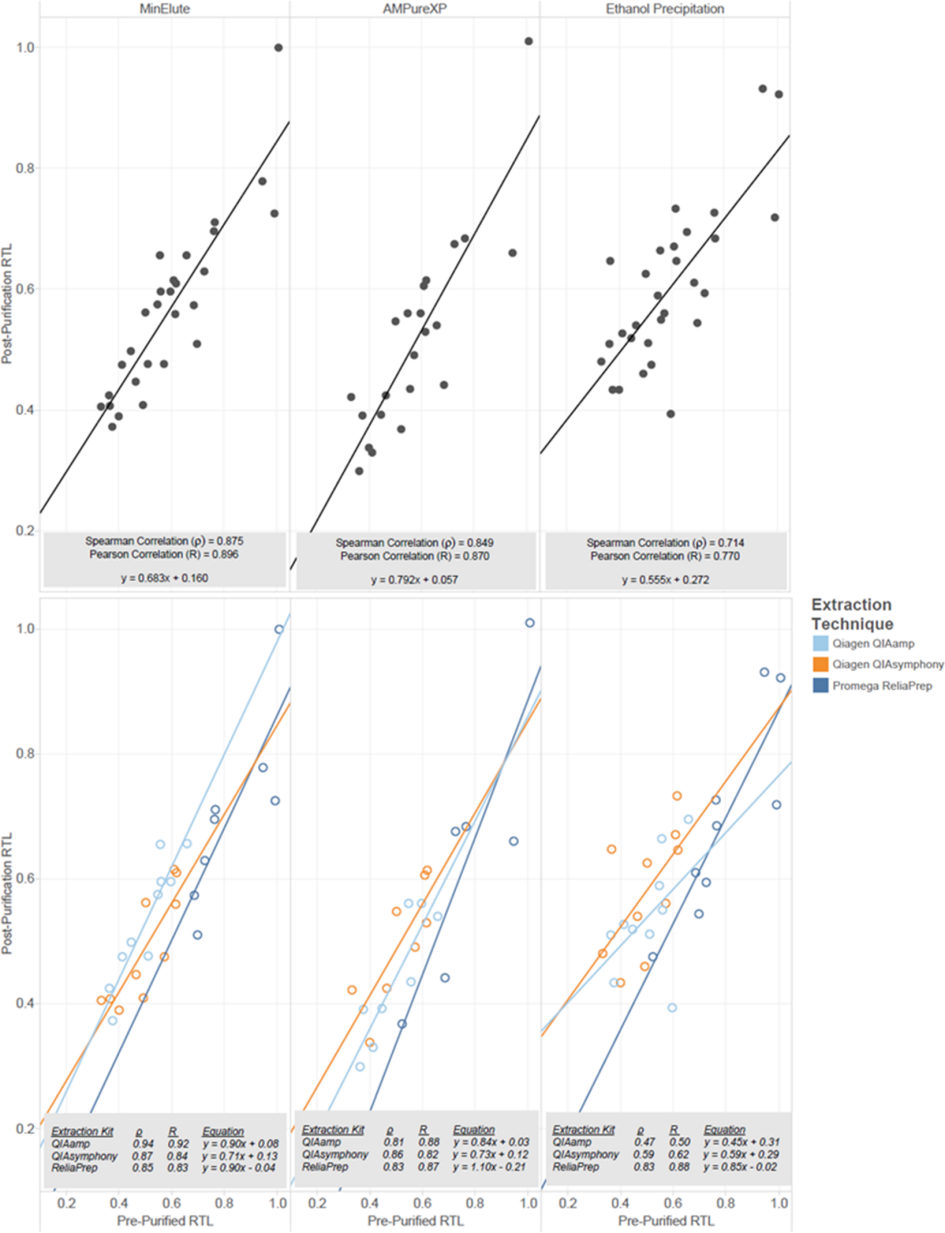
Correlation of relative telomere length (standardized T/S ratio) of matched samples pre- and post-purification. (Top) All samples by purification technique. (Bottom) By purification technique and extraction technique, shown by color, for 10 matched subjects extracted using three different techniques.

The 30 DNA samples, three extraction methods from 10 matched subjects, underwent three different DNA purification methods (S3 Dataset). The CV for internal control replicates (n = 10) standardized T/S ratios was 8.01%. The magnitude of correlation was attenuated (R^2^ = 0.68, ρ = 0.78) after the samples underwent additional purifications. When evaluating each inhibitor removal technique, the correlations of RTL of non-purified DNA to RTL after each purification type change slightly to, R^2^ = 0.80, 0.76, and 0.59 (ρ = 0.88, 0.85, and 0.71) for MinElute, AMPureXP, and EtOH, respectively (Fig 3). The AMPureXP and MinElute techniques maintain a stronger correlation to results prior to purification than those of the EtOH technique, but each purification technique appears to affect the dynamic range differently, as indicated by the slope and intercept differences. The effect of DNA purification technique was independent of the DNA extraction technique for AMPureXP and MinElute. However, the EtOH purification technique for samples extracted via Promega ReliaPrep showed a stronger correlation post-purification (R^2^ = 0.77, ρ = 0.83) than those samples from the same subject extracted via QIAamp (R^2^ = 0.24, ρ = 0.47) and QIAsymphony (R^2^ = 0.38, ρ = 0.59).

### Evaluation of storage temperature and concentration

**Fig 4:**
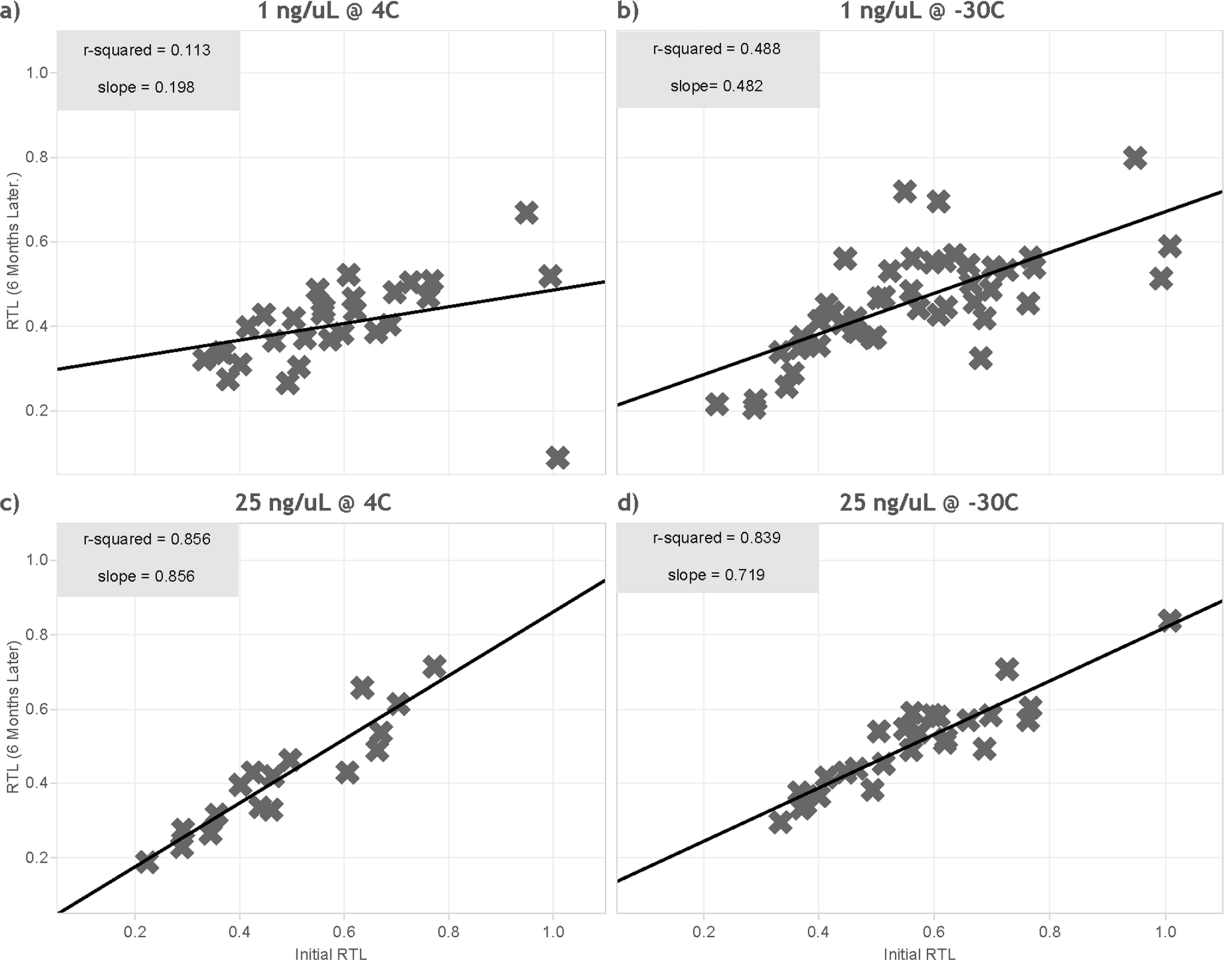
Correlation of relative telomere length (standardized T/S ratio) of same samples after 6 months at various concentrations and storage temperature conditions. (a) 1 ng/uL at 4°C, (b) 1 ng/uL at -30°C, (c) 25 ng/uL at 4°C, and (d) 25 ng/uL at -30°C.

For this experiment, the CV for internal control replicates (n = 10) standardized T/S ratio was 6.03%. Samples stored at 25 ng/uL maintained strong correlations to the original results after 6 months at both 4°C (R^2^ = 0.86) and -30°C (R^2^ = 0.84) storage temperatures. Samples normalized to 1 ng/uL were very weakly correlated to their original results when stored at 4°C (R^2^ = 0.11) and only moderately correlated when stored at -30°C (R^2^ = 0.49) (Fig 4, S4 Dataset).

## Discussion

There is accumulating evidence that certain pre-analytic variables may be important in assuring reliability of the qPCR RTL measurements [12, 17, 21–23, 27]. Here, we assessed some of these variables including extraction technique, inhibitor removal technique, sample storage conditions, and assay plate location. Our results show that DNA extraction method, inhibitor removal techniques, and sample storage conditions significantly contribute to variability in RTL, while location of the sample on the assay plate has no or minor effect as previously observed [28].

Our data further illustrate the importance of using one DNA extraction method for an entire study. Alternatively, a DNA extraction method-specific calibrator could be used if multiple extraction methods are planned, as recently described [26]. We confirmed that the use of different DNA extraction technique introduces variability in RTL measurement. Findings from previous studies evaluated DNA extraction techniques across different methodologies (either solid phase adsorption, magnetic bead adsorption, precipitation, or phenol-chloroform) [21–23]. Here, we show that variability is also present between DNA extraction kits that use the same methodology. Specifically, we compared two different magnetic bead adsorption techniques (ReliaPrep and QIAsymphony) and showed that they not only had different dynamic ranges, but also had poor correlation of RTL in biological replicates.

Our data also demonstrated that variability can also be introduced in RTL by different inhibitor removal (purification) techniques, which are often necessary due to substances intrinsic to specific biological source materials or introduced during extraction or processing [30]. This variability further illustrates the importance of processing all samples within a study in the same manner.

The observed variability in RTL introduced by DNA sample storage temperature and concentration is not surprising as it is known that low concentration solutions of DNA are prone to DNA degradation or other loss over time [31]. However, the extent to which these factors directly affect the telomeric repeat sequences in comparison to that of other regions of the genome, such as the single copy gene (36B4) is not known. This finding also suggests that internal controls utilized for assessment of reproducibility are subject to the same limitations.

In summary, this study shows that pre-analytic factors, including DNA extraction, purification, and storage introduce significant variability in qPCR RTL measurements. Our data show that studies with different pre-analytic methods may not be directly comparable. Therefore, we recommend that these factors be consistent within studies and that multiple replicates within and across studies are used. Researchers should strongly consider validating significant associations between qPCR RTL and disease in a different laboratory and, ideally, with a different measure of TL measurement.

## Supporting information

S1 Dataset Well position for assay reproducibility. Dataset contains 384-well plate descriptor (A, B, C), Well Position, Sample ID, LightCycler instrument number, and standardized T/S Ratio for the experiment regarding well position and assay reproducibility.
(XLSX)

S2 Dataset DNA extraction method. Dataset contains blinded subject ID, extraction method utilized and standardized T/S Ratio for the experiment regarding DNA extraction method.
(XLSX)

S3 Dataset DNA purification technique. Dataset contains blinded subject ID, extraction method utilized, purification technique utilized and standardized T/S ratio for the experiment regarding DNA purification technique and extraction method.
(XLSX)

S4 Dataset Storage temperature and concentration. Dataset contains sample ID, standardized T/S ratio of initial analysis, standardized T/S ratio of analysis post-storage (6 months later), storage temperature (°C) and concentration of DNA (ng/uL) prior to storage.
(XLSX)

## References

[1] O'Sullivan RJ, Karlseder J. Telomeres: protecting chromosomes against genome instability. Nat Rev Mol Cell Biol. 2010;11(3):171–81. doi: 10.1038/nrm2848 ; PubMed Central PMCID: PMCPMC2842081. 20125188 10.1038/nrm2848 PMC2842081

[2] Aubert G, Lansdorp PM. Telomeres and Aging. Physiological Reviews. 2008;88(2):557–79. doi: 10.1152/physrev.00026.2007 18391173 10.1152/physrev.00026.2007

[3] Fitzpatrick AL, Kronmal RA, Gardner JP, Psaty BM, Jenny NS, Tracy RP, et al Leukocyte Telomere Length and Cardiovascular Disease in the Cardiovascular Health Study. American Journal of Epidemiology. 2007;165(1):14–21. doi: 10.1093/aje/kwj346 17043079 10.1093/aje/kwj346

[4] van der Harst P, van der Steege G, de Boer RA, Voors AA, Hall AS, Mulder MJ, et al Telomere Length of Circulating Leukocytes Is Decreased in Patients With Chronic Heart Failure. Journal of the American College of Cardiology. 2007;49(13):1459–64. doi: 10.1016/j.jacc.2007.01.027 17397675 10.1016/j.jacc.2007.01.027

[5] Willeit P, Willeit J, Mayr A, et al Telomere length and risk of incident cancer and cancer mortality. JAMA. 2010;304(1):69–75. doi: 10.1001/jama.2010.897 20606151 10.1001/jama.2010.897

[6] McGrath M, Wong JYY, Michaud D, Hunter DJ, De Vivo I. Telomere Length, Cigarette Smoking, and Bladder Cancer Risk in Men and Women. Cancer Epidemiology Biomarkers & Prevention. 2007;16(4):815–9. doi: 10.1158/1055-9965.epi-06-0961 17416776 10.1158/1055-9965.EPI-06-0961

[7] Armanios M. Syndromes of Telomere Shortening. Annual review of genomics and human genetics. 2009;10:45 doi: 10.1146/annurev-genom-082908-150046 PMC2818564. 19405848 10.1146/annurev-genom-082908-150046 PMC2818564

[8] Harley CB, Futcher AB, Greider CW. Telomeres shorten during ageing of human fibroblasts. Nature. 1990;345:458–60. doi: 10.1038/345458a0 2342578 10.1038/345458a0

[9] Blasco MA. Telomere length, stem cells and aging. Nature chemical biology. 2007;3(10):640–9. doi: 10.1038/nchembio.2007.38 17876321 10.1038/nchembio.2007.38

[10] Alter BP, Rosenberg PS, Giri N, Baerlocher GM, Lansdorp PM, Savage SA. Telomere length is associated with disease severity and declines with age in dyskeratosis congenita. Haematologica. 2012;97(3):353–9. doi: 10.3324/haematol.2011.055269 22058220 10.3324/haematol.2011.055269 PMC3291588

[11] Montpetit AJ, Alhareeri AA, Montpetit M, Starkweather AR, Elmore LW, Filler K, et al Telomere Length: A Review of Methods for Measurement. Nursing Research. 2014;63(4):289–99. doi: 10.1097/NNR.0000000000000037 24977726 10.1097/NNR.0000000000000037 PMC4292845

[12] Aubert G, Hills M, Lansdorp PM. Telomere length measurement—Caveats and a critical assessment of the available technologies and tools. Mutation Research/Fundamental and Molecular Mechanisms of Mutagenesis. 2012;730(1):59–67. 21663926 10.1016/j.mrfmmm.2011.04.003 PMC3460641

[13] Cawthon RM. Telomere measurement by quantitative PCR. Nucleic acids research. 2002;30(10):e47–e. 12000852 10.1093/nar/30.10.e47 PMC115301

[14] Cawthon RM. Telomere length measurement by a novel monochrome multiplex quantitative PCR method. Nucleic acids research. 2009;37(3):e21–e. doi: 10.1093/nar/gkn1027 19129229 10.1093/nar/gkn1027 PMC2647324

[15] O'Callaghan NJ, Fenech M. A quantitative PCR method for measuring absolute telomere length. Biological procedures online. 2011;13(1):1. 10.1186/1480-9222-13-3 PMC3047434 21369534

[16] Elbers CC, Garcia ME, Kimura M, Cummings SR, Nalls MA, Newman AB, et al Comparison between Southern blots and qPCR analysis of leukocyte telomere length in the Health ABC Study. The Journals of Gerontology Series A: Biological Sciences and Medical Sciences. 2014;69(5):527–31. 10.1093/gerona/glt121 PMC4049151 23946336

[17] Martin-Ruiz CM, Baird D, Roger L, Boukamp P, Krunic D, Cawthon R, et al Reproducibility of telomere length assessment: Authors’ Response to Damjan Krstajic and LjubomirButurovic. International Journal of Epidemiology. 2015 doi: 10.1093/ije/dyv170 26403811 10.1093/ije/dyv170

[18] Gutierrez-Rodrigues F, Santana-Lemos BA, Scheucher PS, Alves-Paiva RM, Calado RT. Direct comparison of flow-FISH and qPCR as diagnostic tests for telomere length measurement in humans. PloS one. 2014;9(11):e113747 doi: 10.1371/journal.pone.0113747 25409313 10.1371/journal.pone.0113747 PMC4237503

[19] Brouilette SW, Moore JS, McMahon AD, Thompson JR, Ford I, Shepherd J, et al Telomere length, risk of coronary heart disease, and statin treatment in the West of Scotland Primary Prevention Study: a nested case-control study. The Lancet. 2007;369(9556):107–14. 10.1016/S0140-6736(07)60071-3 17223473

[20] Aviv A, Hunt SC, Lin J, Cao X, Kimura M, Blackburn E. Impartial comparative analysis of measurement of leukocyte telomere length/DNA content by Southern blots and qPCR. Nucleic Acids Research. 2011 doi: 10.1093/nar/gkr634 21824912 10.1093/nar/gkr634 PMC3203599

[21] Cunningham JM, Johnson RA, Litzelman K, Skinner HG, Seo S, Engelman CD, et al Telomere length varies by DNA extraction method. Cancer Epidemiology Biomarkers and Prevention. 2013;22(11):2047–54. 10.1158/1055-9965.EPI-13-0409 PMC3827976 24019396

[22] Hofmann JN, Hutchinson AA, Cawthon R, Liu C-S, Lynch SM, Lan Q, et al Telomere length varies by DNA extraction method: implications for epidemiologic research—letter. Cancer Epidemiology Biomarkers & Prevention. 2014;23(6):1129–30. 10.1158/1055-9965.EPI-14-0145 PMC4051398 24798729

[23] Raschenberger J, Lamina C, Haun M, Kollerits B, Coassin S, Boes E, et al Influence of DNA extraction methods on relative telomere length measurements and its impact on epidemiological studies. Scientific Reports. 2016;6:25398 doi: 10.1038/srep25398 PMC4853716. 27138987 10.1038/srep25398 PMC4853716

[24] Tolios A, Teupser D, Holdt LM. Preanalytical Conditions and DNA Isolation Methods Affect Telomere Length Quantification in Whole Blood. PloS one. 2015;10(12):e0143889 doi: 10.1371/journal.pone.0143889 26636575 10.1371/journal.pone.0143889 PMC4670203

[25] Denham J, Marques FZ, Charchar FJ. Leukocyte telomere length variation due to DNA extraction method. BMC research notes. 2014;7(1):877. 25475541 10.1186/1756-0500-7-877 PMC4289347

[26] Seeker LA, Holland R, Underwood S, Fairlie J, Psifidi A, Ilska JJ, et al Method specific calibration corrects for DNA extraction method effects on relative telomere length measurements by quantitative PCR. PloS one. 2016;11(10):e0164046 doi: 10.1371/journal.pone.0164046 27723841 10.1371/journal.pone.0164046 PMC5056729

[27] Koppelstaetter C, Jennings P, Hochegger K, Perco P, Ischia R, Karkoszka H, et al Effect of tissue fixatives on telomere length determination by quantitative PCR. Mechanisms of ageing and development. 2005;126(12):1331–3. doi: 10.1016/j.mad.2005.08.003 16182339 10.1016/j.mad.2005.08.003

[28] Eisenberg DTA, Kuzawa CW, Hayes MG. Improving qPCR telomere length assays: Controlling for well position effects increases statistical power. American Journal of Human Biology. 2015;27(4):570–5. doi: 10.1002/ajhb.22690 25757675 10.1002/ajhb.22690 PMC4478151

[29] Callicott RJ, Womack JE. Real-time PCR assay for measurement of mouse telomeres. Comparative medicine. 2006;56(1):17–22. 16521855

[30] Schrader C, Schielke A, Ellerbroek L, Johne R. PCR inhibitors–occurrence, properties and removal. Journal of applied microbiology. 2012;113(5):1014–26. doi: 10.1111/j.1365-2672.2012.05384.x 22747964 10.1111/j.1365-2672.2012.05384.x

[31] Röder B, Frühwirth K, Vogl C, Wagner M, Rossmanith P. Impact of long-term storage on stability of standard DNA for nucleic acid-based methods. Journal of clinical microbiology. 2010;48(11):4260–2. doi: 10.1128/JCM.01230-10 20810770 10.1128/JCM.01230-10 PMC3020885

